# Morphological Effects in SnO_2_ Chemiresistors for Ethanol Detection: A Review in Terms of Central Performances and Outliers

**DOI:** 10.3390/s21010029

**Published:** 2020-12-23

**Authors:** Andrea Ponzoni

**Affiliations:** Unit of Brescia, National Institute of Optics of the National Research Council (CNR-INO), 25123 Brescia, Italy; andrea.ponzoni@ino.cnr.it

**Keywords:** chemiresistors, SnO_2_, ethanol, nanoparticles, nanorods, nanosheets, hierarchical nanostructures

## Abstract

SnO_2_ is one of the most studied materials in gas sensing and is often used as a benchmark for other metal oxide-based gas sensors. To optimize its structural and functional features, the fine tuning of the morphology in nanoparticles, nanowires, nanosheets and their eventual hierarchical organization has become an active field of research. In this paper, the different SnO_2_ morphologies reported in literature in the last five years are systematically compared in terms of response amplitude through a statistical approach. To have a dataset as homogeneous as possible, which is necessary for a reliable comparison, the analysis is carried out on sensors based on pure SnO_2_, focusing on ethanol detection in a dry air background as case study. Concerning the central performances of each morphology, results indicate that none clearly outperform the others, while a few individual materials emerge as remarkable outliers with respect to the whole dataset. The observed central performances and outliers may represent a suitable reference for future research activities in the field.

## 1. Introduction

Metal oxides (MOXs) are among the widest investigated materials in the gas sensing field. This is thanks to their capability to exhibit large electrical resistance variations upon exposure to low concentrations of different chemicals, and to the availability of cheap synthesis methods compatible with production at large scale [1,2]. Moreover, their reduced size, weight and power consumption, merged with their compatibility with silicon technology, makes MOX-based chemiresistors ideal candidates for the development of portable devices [3,4,5].

Their effectiveness has been proven in many applicative fields, including medicine [6,7], environmental monitoring [8,9], food processing and quality control [10,11], safety and security [12,13]. 

Several MOX materials were successfully exploited as gas sensors, including for example WO_3_, ZnO, In_2_O_3_, TiO_2_, but the largest amount of work has been done using SnO_2_. In particular, the first commercial MOX chemiresistor was based on a SnO_2_ thick film, i.e., a disordered network composed of crystallites with spherical shape, [2] and, along years, several milestones in the understanding of the MOX sensing mechanism have been achieved working with SnO_2_ thick films, which is hence considered the reference material in the field [14,15].

In addition to the choice of the base MOX material, different strategies have been employed to tune and optimize the sensing capability of MOX thick films toward specific applications. These strategies include the use of dopants inside the MOX lattice [16,17], the dispersion of inorganic catalysts or organic layers over the MOX surface [1,18], the use of mixed oxides [19,20], the fine tuning of the film morphology [21,22]. Concerning the latter, a variety of methods have been developed in the last years to control the morphology at different levels, from the shape of elementary building blocks till their eventual assembly in hierarchical structures. Materials such as nanowires, nanorods, nanosheets, as well as hierarchical structures including, for example, hollow spheres, fibers, flowers, have become the focus of intense research [23,24,25].

In this context, the present paper aims to review the different morphologies developed in the last years and compare them through a systematic analysis of the reported papers, extrapolating the mean performance of each morphology, where ‘mean’ is not necessarily the arithmetic mean, but it stands for the most appropriate parameter that expresses the central behavior of the considered class. Once established such an appropriate parameter, emphasis is given to those materials that emerges as outperforming the mean. In order to have a dataset as homogeneous as possible, which is necessary for a comparative analysis, the review is focused on pure SnO_2_ as target material and ethanol as target molecule, tested in a dry air background. The choice of SnO_2_ is because it is the widest investigated metal oxide material, hence it offers the largest statistics, and because, in line with the tradition of gas sensing, SnO_2_ is often adopted as benchmark material for other MOX [14,26]. Getting its central performance and identifying outliers may hence provide a useful reference for future works on MOX-based gas sensors in general, not only limited to SnO_2_ itself. The choice of ethanol is because its importance as basic chemical in many applicative fields [27,28,29], which make it a widely investigated molecule to test sensor materials and morphologies [30]. 

The rest of the manuscript is organized in four sections (numbered from 2 to 5): the former (Section 2) provides a resume of the working mechanism of MOX chemiresistors, with emphasis on morphological effects. Details about the procedure adopted to perform the statistical analysis are reported in Section 3, results of the statistical analysis are presented in Section 4, these are compared with findings reported in individual papers in Section 5, finally conclusions are in Section 6.

## 2. Sensing Mechanism and Morphology Effects

### 2.1. Interaction between Ethanol and SnO_2_

The working mechanism of MOX chemiresistors is based on the modulation of their electrical conductance as a consequence of interaction with gases. At molecular level, the interaction mainly occurs with active ions such as *OH^−^*, *O_2_^−^*, *O^−^*, *O^2−^* that cover the MOX surface [14].

Ethanol detection is typically optimized at temperatures between 250 and 400 °C. In this range, *O^−^* is the dominating active ion and its chemisorption from the atmosphere is described by the following reaction [31]:

(1) $$O_{2}+2e^{-}⇄2O_{}^{-}$$

The further reaction of *O^−^* with ethanol is often reported in gas-sensing literature as follows [32,33]:

(2) $$CH_{3}CH_{2}OH+6O^{-}\;\to \;2CO_{2}+3H_{2}O+6e^{-}$$

A deeper analysis of ethanol oxidation over metal oxide surfaces shows that Equation (2) may take place according to two main routes, namely dehydrogenation into acetaldehyde or dehydration into ethylene, whose intermediates are finally oxidized to *H_2_O* and *CO_2_* [34,35]. 

**Figure 1:**
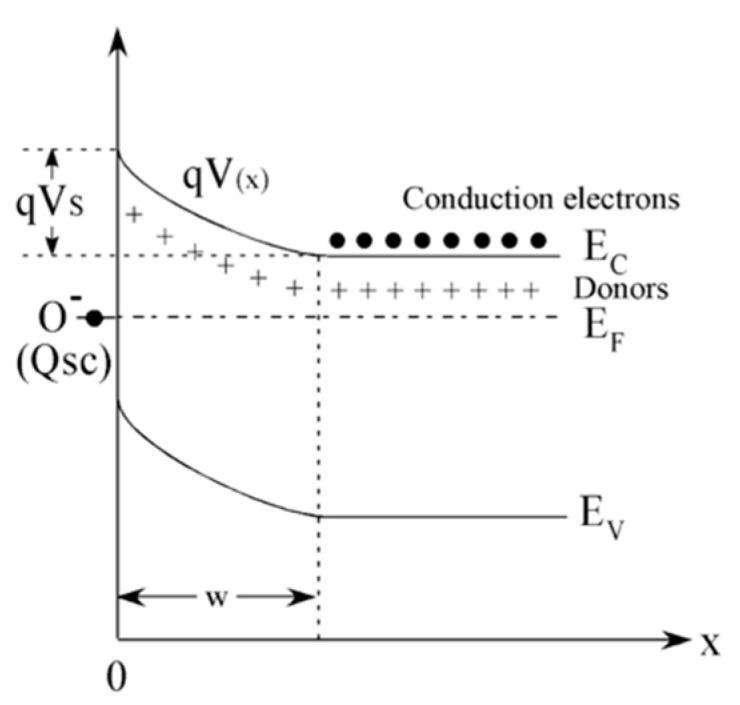
Schematic representation of the energy band diagram of an n-type semiconductor, such as SnO2, upon chemisorption. E_f_, E_c_, E_v_ represent the Fermi energy, the edge of the valence band of the semiconductor and of the edge of its conduction band, respectively. qV_S_ is the Schottky barrier developed at the surface and Qsc the charge trapped at the acceptor surface states created by oxygen chemisorption. W is the depletion layer. Reprinted from [38], Copyright (2008), with permission from Elsevier.

From the semiconductor viewpoint, the oxygen chemisorption process creates acceptor surface states that withdrawn electrons from the SnO_2_ conduction band. By combining the electrical charge neutrality in the semiconductor with the chemical equilibrium at the surface, the chemisorption process results in the establishment of a surface layer that, in the abrupt approximation, is fully depleted of electrons. This redistribution of charges induces an electric field that bands the band structure of the semiconductor raising a Schottky barrier at the surface as schematically shown in Figure 1 [14,31,36]. Reducing gases, such as ethanol, modulate the electrical properties of MOX materials by reducing the population of chemisorbed oxygen ions according to Equation (2) and, in turn, the depth of the depletion layer and the Schottky barrier height [14,37].

The width W of the depletion layer and the surface potential V_S_ are related one another through Equation (3):

(3) $$W=\sqrt{\frac{2{\varepsilon V}_{S}}{{\mathrm{qn}}_{b}}}$$

where q is the electron charge, ε and n_b_ are the permittivity and the charge carrier density of the bulk semiconductor [14].

This model is suitable to understand the behavior of many experimental results reported in literature about MOX-based gas sensors. For example, the empirical power law describing the calibration curve, Equation (4), may be retrieved in this framework [38]:

(4) $$\frac{G_{\mathrm{gas}}}{G_{\mathrm{air}}}=1+{\mathrm{AC}}^{Z}$$

where *C* is the ethanol concentration (in ppm), A and Z are fitting parameters retrieved case by case. In particular, the model shows that A depends on the reaction rates of Equations (1) and (2) promoted by the MOX material, while Z depends mainly from the dominant oxy-ion. Compounds undergoing complex reactions, involving for example different pathways or intermediate by-products, may exhibit slight variations in Z [38]. Additional effects explained in the framework of this model are size and shape effects, which are reported in Section 2.2 and Section 2.3, respectively.

### 2.2. Crystallite Size Effects

The model described in the previous sections works until the width of the depletion layer, W, does not extend through the whole volume of the crystallite. Considering crystallites with spherical shape and diameter t, the model works for t > 2W. In these conditions, which are typically referred as ‘the regional depletion regime’, the interaction with gases modulates the electrical properties of the MOX crystallites only in the surface region and the Schottky barrier controls both the electrical transport across grains and the response to gases. The electrical resistance R is thermally activated with the activation energy given by qV_S_ and the response intensity to gases is almost independent from the grain size [15,37]:

(5) $$R\propto \exp\left(\frac{{\mathrm{qV}}_{S}}{k_{B}T}\right)$$

where T denotes the sensor temperature in Kelvin degrees and k_B_ the Boltzmann constant.

On the other hand, if grains are small or the gas concentration is such that t would be smaller than the resulting 2W, crystallites will be (almost) fully depleted of electrons, and the band diagram profile will lie above the energy of the bulk conduction band, E_c_, all over the entire grain. In this regime, called ‘the volume depletion regime’, the electrical resistance is still thermally activated but its activation energy E_A_ is no more the Schottky barrier, which is much lower than in the previous case, but the energy to promote charge carriers in the raised conduction band, which is nearly constant thorough the whole volume of the grain, [31,39]:

(6) $$R\propto \exp\left(\frac{E_{A}}{k_{B}T}\right)$$

In this regime, the response intensity to gases will increase with decreasing t, [15,37]. This provides the theoretical framework to explain the benefit arising from the use of nanostructured materials, which is widely observed in experiments since several years and has become a leading concept in the design of sensitive layers.

**Figure 2:**
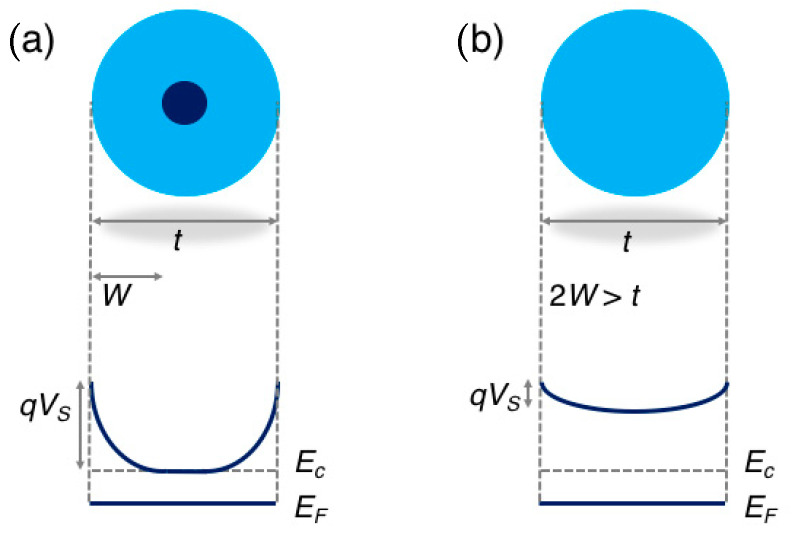
Schematic representation of the energy band diagram in a spherical grain of diameter t in the regional depletion regime (**a**), in which the grain diameter is larger than two times the depletion layer W (t > 2W), and in the volume depletion regime (**b**), in which 2W > t. Adapted with permission from [40]. Copyright (2018) American Chemical Society.

The band diagram in the two regimes is schematically reported in Figure 2.

Considering the typical parameters of SnO_2_, ε ≈ 10^−10^ F/m and n_b_ ≈ 5 × 10^18^ cm^−3^ [41,42], according to Equation (3), the depletion layer results $W\approx 1.6\times 10^{-8}\sqrt{V_{S}}$, which means W ≈ 12 nm for a typical value of V_S_ ≈ 0.65 V measured in air at a sensor temperature around 350 °C [43].

### 2.3. Crystallite Shape Effects

Films traditionally employed in gas sensing were composed by disordered assemblies of spherical (or nearly spherical) crystallites, hence most of models about MOX gas sensors have been developed referring to this kind of film [14,15,38,39]. With the advent of new morphologies such as nanowires and nanosheets and the increasing number of papers reporting the experimental investigation of their sensing properties, attention has been dedicated to these new nanostructures also from a theoretical point of view [31,37,44]. For example, the dependence of the band bending profile in Equation (6) has been solved for the spherical, cylindrical and slab-like shape of crystallites [31,37,44]. 

Using the term crystallite thickness, t, to refer to the smallest dimension of each crystallite, namely the diameter for spherical and cylindrical crystallites and the thickness itself for slab-shaped crystals and comparing crystallites with the same thickness, these models provide the following information [31,44]:

(1) Under the same gas exposure conditions, the width of depletion layer is shape dependent and decreases with the following order: nanoparticles—nanorods—nanosheets;
(2) Increasing the concentration of an oxidizing specie such as oxygen, which means increasing *qV_S_*, crystallites enters in the full-depletion regime in the following order: nanoparticles—nanorods—nanosheets;
(3) The crystallite shape weakly affect the exponent Z of the power law expressed by Equation (4), which is hence mainly determined by the dominant oxy-ion involved in the reaction and eventually by the complexity of the reaction itself (see Section 2.1).

Based on points (1) and (2), spherical crystallites appears as more efficient transducers with respect to their sheet- and wire-shaped counterparts.

### 2.4. Gas Diffusion through the Sensing Film

Models summarized in previous sections describes the gas-sensing mechanism at the level of microscopic crystallites. 

In order to have a macroscopic film suitable to properly exploit a network of finely tuned microscopic elements, it is necessary that gas molecules have easy access to as many crystals as possible through the whole thickness of the sensing layer. 

To achieve this result, two important morphological features should be realized: (i) contacts between neighboring crystallites should be as small as possible, avoiding the formation of compact aggregates, in which gas hardly diffuses hence leaving crystallites located at the center of the agglomerate almost unreached by gas molecules; (ii) the spatial arrangement of crystallites should leave pores large enough to allow an ease diffusion of gas molecules through the whole volume of the film. This latter feature is even more important considering that the target gas is consumed by the interaction with the MOX surface. As a consequence, the gas concentration will decrease with moving to deeper layers of the sensing film. If an efficient reaction with the gas is not accompanied by a structure offering the necessary diffusion, the interaction with the target gas may be limited to the upper portion of the sensing material, the lower layers remaining unreached by the target gas.

The concentration profile of the target gas inside the sensing film and the response intensity dependence from the film thickness L were calculated by Sakai and coworkers [45] under the hypothesis of a linear calibration curve, Z = 1 in Equation (4), and are reported in Equations (7) and (8) respectively:

(7) $$C_{x}=\;C\frac{\cos h\left(1-x/L\right)}{\cos h\left(m\right)};\;m=L\sqrt{\frac{k}{D_{k}}}$$

(8) $$\frac{G_{\mathrm{gas}}}{G_{\mathrm{air}}}=1+A\frac{\tan h\left(m\right)}{m}C;\;m=L\sqrt{\frac{k}{D_{k}}}$$

Here x is the distance from the surface through the film thickness, C is the gas concentration in the environment, k is the rate constant of the reaction that consumes the target gas, such as the reaction reported in Equation (2), A has the same meaning as in Equation (4), D_k_ is the Knudsen diffusion coefficient, $D_{k}=\;\frac{4r_{p}}{3}\sqrt{2\mathrm{RT}/\left(\pi M\right)}$, where r_p_ is the average pore radius, R is the gas constant, M the molecular mass of the target gas molecule and T the sensor temperature in degrees Kelvin. 

A detailed discussion of the meaning of these equations is provided in [45]. For the purpose of the present paper, it is worth mentioning the dependence from the k/D_k_ ratio, i.e., the importance of large pores, especially in the case of efficient reactions (large k).

In view of these arguments, it is worth mentioning that though nanoparticles appear more efficient than nanowires and nanosheets at the level of elementary building blocks, as discussed in Section 2.3, very thin nanoparticles often leave small voids, which, in turn, hinder an efficient diffusion of molecules. Moreover, thin nanoparticles are widely reported to suffer coalescence effects, especially at the high working temperatures of MOX chemiresistors. This may lead to the formation of large aggregates, which loose the efficiency of the original nanoparticles. In this sense, nanowires and nanosheets are more effective in the realization of macroscopic layers featuring an open structure with large pores [46].

## 3. Materials and Methods

The analysis has been performed on chemiresistors based on pure SnO_2_, i.e., SnO_2_ materials that are neither intentionally doped nor intentionally functionalized with any catalyst, choosing ethanol as target chemical. Given the well-known effect of humidity on sensing performance [16,47], in order to compare the devices in conditions as much similar as possible, only sensors tested in a dry air background were considered. 

Qualitative and quantitative descriptors adopted to characterize the morphological features of the reviewed materials are described in Section 3.1, while functional parameters used to evaluate the sensor performance are in Section 3.2.

### 3.1. Morphological Classes and Descriptors

To investigate morphological effects on the sensor performance, literature materials have been grouped based on the shape of their elementary building blocks (crystallites) and their eventual hierarchical assembly.

**Figure 3:**
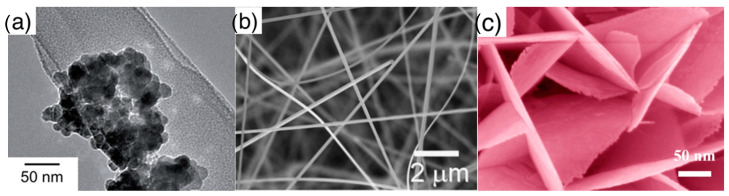
Elementary units (crystallites) composing the nanostructured metal oxide layers: (**a**) nanoparticles, (**b**) nanorods, (**c**) nanosheets. (**a**) is reprinted with permission of Royal Society of Chemistry (RSC), from [16], permission conveyed through Copyright Clearance Center, Inc.; (**b**) is reprinted from [48], Copyright (2008), with permission from Elsevier; (**c**) is reprinted with permission from [49], Copyright (2014) American Chemical Society.

**Figure 4:**
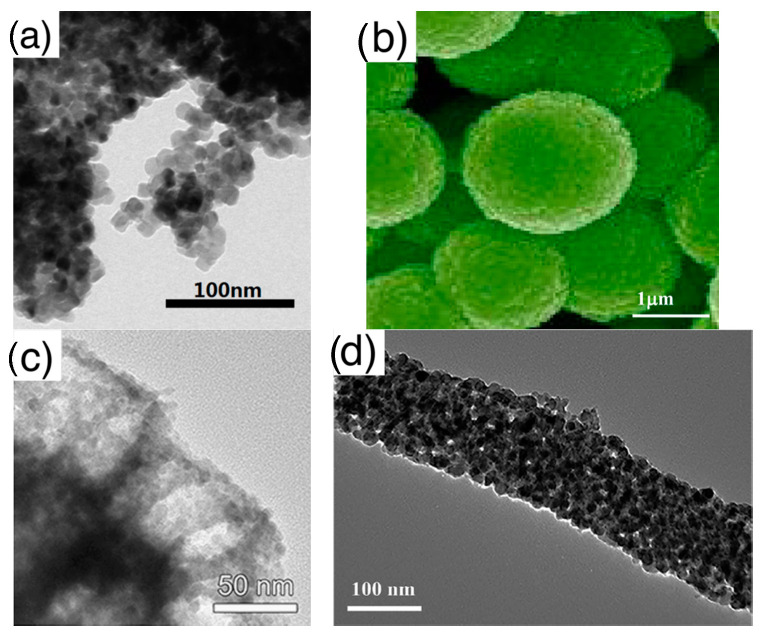

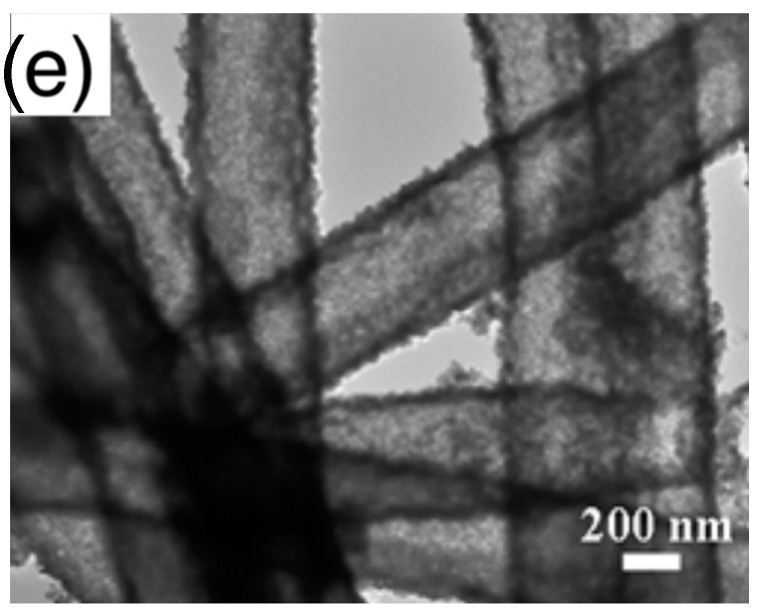
Types of elementary units assemblies (using nanoparticles as elementary building blocks): (**a**) disordered nanoparticles network with no hierarchical organization; (**b**) networks of nanoparticles organized in spherical assemblies; (**c**) nanoparticles organized in porous spheres assemblies, detail of a single porous sphere; (**d**) networks of nanoparticles organized in fiber-like assemblies, zoom over a single fiber; (**e**) networks of nanoparticles organized in porous-fiber assemblies. (**a**) is reprinted from [50] Copyright (2016), with permission from Elsevier; (**b**) is reprinted from [51] Copyright (2013), with permission from Elsevier; (**c**) is reprinted from [52]; (**d**) fiber is reprinted from [53] Copyright (2017), with permission from Elsevier; (**e**) is reprinted from [54] Copyright (2015), with permission from Elsevier.

- Elementary building blocks (shape of): the first classification is done in terms of shape of elementary crystallites composing the material, classifying it according to three common types as listed below and shown in Figure 3:
  ◦ Nanoparticles: crystallites with spherical shape, these are the typical elementary units of traditional thick films, which are also widely studied nowadays. With respect to other crystallites, nanoparticles, due to their rounded shape, do not feature a clear surface termination in terms of crystalline planes;
  ◦ Nanorods: crystallites featuring an elongated shape, with surfaces usually identified by well-defined crystalline planes. This class comprises also crystallites identified in literature as nanowires or nanobelts. Crystals with cubic, octahedral or elongated octahedral shape are also included in the nanorods class due to the common feature of faceted surface;
  ◦ Nanosheets: single crystalline, thin nanostructures extending in two dimensions. Nanostructures named in literature nanoplates, nanoplatelets, nanolamellae, nanodiscs are also included in this class.
- Assembly (of elementary building blocks): the macroscopic sensing layer is formed by a disordered network. Crystallites may be the components of this network or, in some cases, they are organized to form larger assemblies, which in turn, compose the disordered network. The different assemblies investigated in literature are grouped in the following five classes, for which Figure 4 reports examples based on nanoparticles as elementary crystallites:
  ◦ Disordered networks with no hierarchical assembly: this is the simplest network, in which elementary nanostructures form a disordered network with no hierarchical organization. The traditional thick film studied in gas sensor belong to this class;
  ◦ Network of fibers: elementary nanostructures are organized to form elongated agglomerates with a compact character, which, in turn, form a disordered network;
  ◦ Network of porous fibers: elementary nanostructures are organized to form elongated agglomerates with a clear porous structure, such as, for example, hollow fibers;
  ◦ Network of spheres: elementary nanostructures form hierarchical structures with compact, spherical shape. Flower like assemblies are included in this group;
  ◦ Network of porous spheres: elementary nanostructures form hierarchical structures with open, porous spherical shape. Hollow spheres are a particular example of this hierarchical morphology.

Overall, the statistical results reported in Section 4 are retrieved based on 121 pure SnO_2_ materials reported in 85 literature articles, whose distribution between the considered morphological classes is summarized in Table 1**,** together with the lists of the respective references. The statistics is highly inhomogeneous with respect to crystallites’ shape and hierarchical assembly. Though the mentioned inhomogeneity may be undesired from a statistical point of view, it is anyway a matter of fact reflecting the larger amount of studies that have been dedicated in the considered period of time to a given morphology with respect to another. 

Morphological characteristics were also evaluated considering the following quantitative descriptors (wherever reported in the respective papers):

- Crystallite thickness, t, which represent the minimum length of crystallites, i.e., the diameter for grains with spherical or wire-like shape and the thickness itself for nanosheets, as determined from X-ray diffraction (XRD) or transmission electron microscopy (TEM) measurements;
- Specific Surface Area, SSA, as determined from Brunauer–Emmett–Teller (BET) analysis of N_2_ adsorption measurements;
- Pore diameter, r_p_, is the mode of the pore distribution as determined through the Barrett–Joyner–Halenda (BJH) analysis of N_2_ desorption measurements.

### 3.2. Parameters Adopted to Quantify the Sensors Performances

The sensing performances of the considered materials were evaluated using the following response parameters:

- Response amplitude, calculated as G_gas_/G_air_, where G_gas_ and G_air_ stands for the steady state conductance measured during gas exposure and in the dry air background, respectively. Concerning those papers reporting the response amplitude as the normalized conductance variation, i.e., (G_gas_ − G_air_)/G_air_, their values were converted in G_gas_/G_air_ using the relationship: (G_gas_ − G_air_)/G_air_ + 1 = G_gas_/G_air_.
- Calibration curve, given by the power-law described by Equation (4), G_gas_/G_air_ = 1 + AC^Z^, from which the parameters A and Z are extrapolated by applying a linear fit based on Least Squares algorithm to ‘Log(G_gas_/G_air_ − 1) vs Log(C)’ data.

## 4. Statistical Analysis

### 4.1. Response Amplitude and Calibration Curve

**Figure 5:**
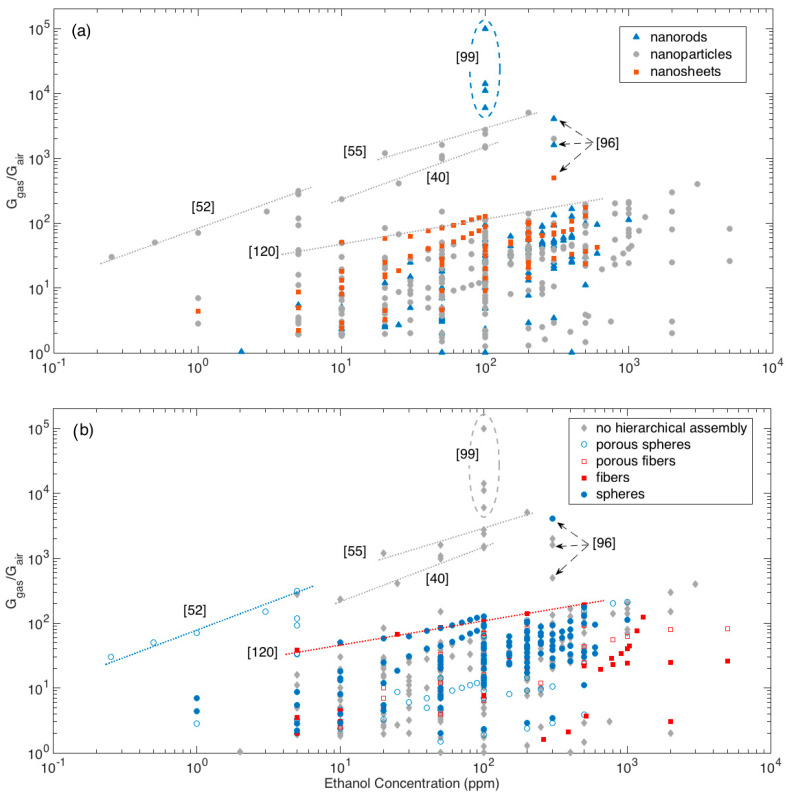
Response amplitude vs ethanol concentration plot, different colors highlights the dependence from elementary building blocks (**a**) and from the assembly of elementary building blocks (**b**). For some particular data-points, the related references are also identified to support the discussion (see text for details).

The whole dataset of response amplitude vs gas concentration analyzed is shown in Figure 5, using different colors and symbols to identify the morphologies of crystallites, Figure 5a, and hierarchical assemblies, Figure 5b.

Most of responses, whichever the crystallite shape and their eventual hierarchical organization, lies below the visual line traced extending the responses (calibration curve) recorded by Li et al. with the nanoparticle-shaped crystallites organized in fiber-like assemblies [120]. Above this line, a few materials outperforming others clearly emerge. 

Among these latter materials, the first mention is for the nanorod networks prepared by Kida and co-workers, which feature the largest responses at all [99]. Specifically, four different networks were prepared, which featured responses of about 6000, 11,000, 14,000 and 100,000 to 100 ppm of ethanol. Responses were observed to increase with increasing the average pore diameter of the sensing layer and ascribed to a more efficient diffusion promoted by the larger pores [99].

Other remarkable responses have been reported for different nanoparticle networks with no hierarchical organization [16,40,55,86]. Visually extrapolating the calibration curves of the reported materials allows to compare layers tested in different concentration ranges. In this sense, the performances recorded for the mentioned nanoparticle networks may be considered comparable with those reported by Lee and coworkers for nanoparticles assembled in porous spheres with multimodal porosity [52] and by Khodadadi and coworkers for spherical assemblies of nanorods [96].

**Figure 6:**
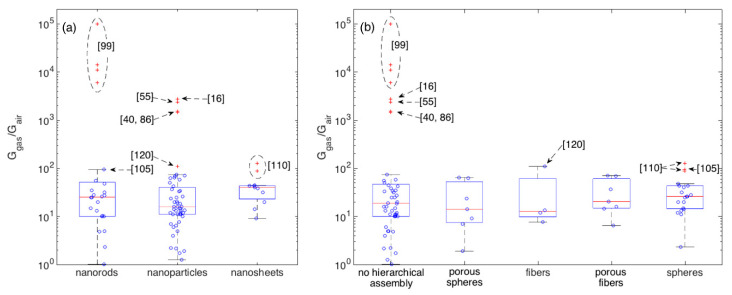
Boxplots showing the statistics of the response amplitude to 100 ppm of ethanol with respect to crystallite morphologies (**a**) and their eventual hierarchical assembly (**b**). Boxplots shows the following parameters: median (central red-mark), 1st and 3rd quartiles (bottom and top box-edges), extreme data not considered outliers (whiskers ends), outliers are individually reported as red crosses, other data-points are visualized as blue circles.

To better get the central performance of each morphological class and appreciate the deviation of outliers with respect to such mean behavior, boxplots are reported in Figure 6 for crystallite (a) and assembly (b) shapes. 100 ppm is here chosen as the reference concentration owing to its larger statistics with respect to other concentrations. Boxplots are tools commonly employed in explorative data analysis [47,127,128]. They report the 2nd quartile, Q2, i.e., the median, as the central red line, 1st and 3rd quartiles, Q1 and Q3, as bottom and top box edges and the two extreme data not considered outliers as whiskers ends. Values being at least 1.5 times the interquartile range beyond the corresponding hinge are considered outliers and are individually reported as red crosses. Non-outliers data-points are reported as blue circles. Statistical parameters describing the distributions of each morphology are reported in Appendix A (Table A1 and Table A2). Several morphologies are characterized by an appreciable number of outliers and, in some cases, only a reduced amount of data is available. Moreover, most of morphologies are characterized by an asymmetric distribution, as indicated, for example, by the skewness and by the difference between the median and the arithmetic mean values. In several cases, the Smirnov-Kolmogorov test return a *p*-value that is lower than the widely adopted threshold of 0.05, supporting the evidence to reject the hypothesis of data following a normal distribution. Given these arguments and considering the need for a uniform approach to describe and compare the central performances of these distributions, the median is considered a more robust indicator than the arithmetic mean [129]. 

In terms of central behavior, no morphological class clearly emerges with respect to others. This is further supported by the median tests, which return *p* > 0.05 for most of comparative tests between couples of morphologies (Appendix A), suggesting the lack of evidence to reject the hypothesis of distribution having comparable medians. The only exception, is the median test between nanoparticles and nanosheets, which returns *p* ≈ 0.02. Though this would suggest to reject the null hypothesis, i.e., to consider the difference between the two distributions large enough to support the existence of a true difference between the two medians, to properly evaluate this conclusion it’s worth considering the effect of outliers. If we re-apply the test after removing the outliers observed in Figure 6, we get *p* > 0.05 for all the tests, with the nanoparticle-nanosheet comparison still returning anyway the smallest *p*-value, specifically *p* ≈ 0.07. Besides the specific values of *p*, the general situation depicted by Figure 6 and related tables is about the evidence of a few outliers that clearly outperform other materials and much weaker differences among the central performances of different morphologies. 

The largest group of outliers is observed for materials sharing the lack of any hierarchical assembly [16,40,55,86,99]. Responses measured in these papers are much larger than those observed in all other papers. Within this outstanding group, the most performing materials have rod-shaped crystallites (with no hierarchical organization), [99], the rest is composed by nanoparticles [16,40,55,86]. Concerning other outliers not in this group, the two types of nanosheets with spherical hierarchical organization developed in [110] are outliers within both the respective crystallite and assembly classes. The difference between these two nanosheet-based layers relies in the sheet width, which are around 400 and 800 nm respectively, while the thickness is similar for both samples, t ≈ 50 nm. Similar responses (G_gas_/G_air_ ≈ 100 to 100 ppm of ethanol) were also recorded with hierarchical fibers composed by nanoparticles [120] and with rod-like nanostructures organized in spherical assemblies [105]. 

To look for other outperforming materials that were not tested at this ethanol concentration, a similar analysis was carried out for other concentrations, namely 10, 50 and 200 ppm, which also feature an appreciable statistics of samples. The 100 ppm outliers, emerges as remarkable outliers also for all other concentrations at which they were measured. As for other materials, a few additional are identified as outliers with responses larger or comparable (depending the considered concentration) than the response recorded in [120]. These are the disordered nanoparticle networks reported in [60,74].

Besides the useful information retrieved from boxplots, they consider one concentration per time, hence the comparison of layers tested at different concentration is not easy, especially for those papers whose concentration ranges do not overlap. In this view, it is worth exploring sensor data set through their calibration curves, which offer the opportunity of a lesser concentration-constrained comparison. 

The calibration curve is a fundamental feature for any given device and it is also the basis for determining additional fundamental parameters such as the limit of detection (LOD) and the sensitivity. The latter, according to the definition provided by the International Union of Pure and Applied Chemistry (IUPAC), is the derivative of the calibration curve. 

According to Equation (4), the calibration curve of MOX chemiresistors is expressed by means of two parameters, A and Z. A large value of A is beneficial for LOD and sensitivity, while these parameters feature different dependences from Z. Indeed, a larger Z means larger sensitivity but also a worse LOD due to the faster decrease of the sensor response with decreasing gas concentration.

**Figure 7:**
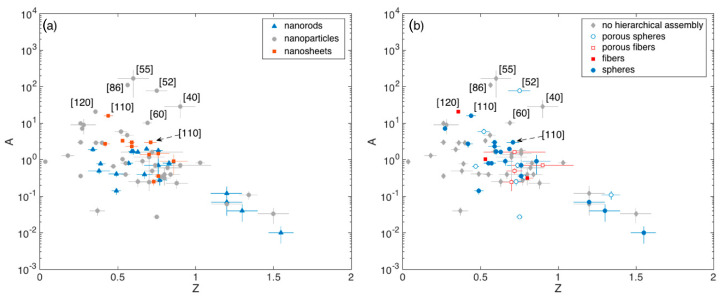
(**a**) Pre-exponential factor A of the calibration curve vs exponent Z extrapolated from fit with Equation (4), with ethanol concentration expressed in ppm. Dependence from crystallite morphology (**a**) and crystallite eventual hierarchical assembly (**b**). In some cases, error bars associated to the fitting parameters are smaller than the size of the respective data symbol.

The A vs Z plots are reported in Figure 7a,b, highlighting the crystallite and assembly morphology of the related materials, respectively. 

These plots show that Z typically lies between 0.5 and 0.8 (Q1 and Q3 respectively), with a median value of about 0.7. The spread is reasonably due to the different optimal temperatures identified in different papers and to the manifold reaction path that ethanol may undergo with SnO_2_, which may induce slight modifications in Z (Section 2.1). 

Outliers identified in Figure 6 and in the related boxplot discussion ([40,55,60,86,110,120]) are all characterized by large A values (A > 10), while Z varies within the aforementioned interquartile range. The porous spheres with multimodal porosity developed by Lee and coworkers [52], lie in this range of outperforming materials also in terms of the A parameter, in agreement with the discussion about Figure 5. Unfortunately, other remarkable materials discussed in Figure 5, namely those reported in [16,74,96,99], were tested against a single concentration of ethanol and it is not possible to retrieve the related calibration curves. 

Based on this analysis it is anyway possible to identify other remarkable materials that were tested against a single ethanol concentration different from 100 ppm. This is the case of the hollow spheres assemblies of nanoparticles reported in [113] and [118], which feature response of about 93 and 33 to 5 ppm of ethanol, which competes well with the responses of about 38 reported in [120], identified as outlier. Remarkable results were also achieved in [69] by using a disordered network of nanoparticle response. The response reported by these authors (≈2000 to 300 ppm) is comparable with the response exhibited by the outliers developed in [96], (Figure 5).

### 4.2. Crystallite Thickness, Specific Surface Area and Pore Radius

The performance of a sensitive layer depends on many physical and chemical parameters and models resumed in Section 2 aims at rationalizing the dependence of the sensor response from a set of these material properties. The crystallite thickness (t) and the Specific Surface Area (SSA) are among the most considered properties to explain the sensing capability of a given layer. Their importance is so marked that they often shadow the effects of other properties, with materials optimized in various ways, for example by addition of dopants or surface catalysts, exhibiting a strict correlation between the response intensity and the larger SSA or the lower t [78,130].

**Figure 8:**
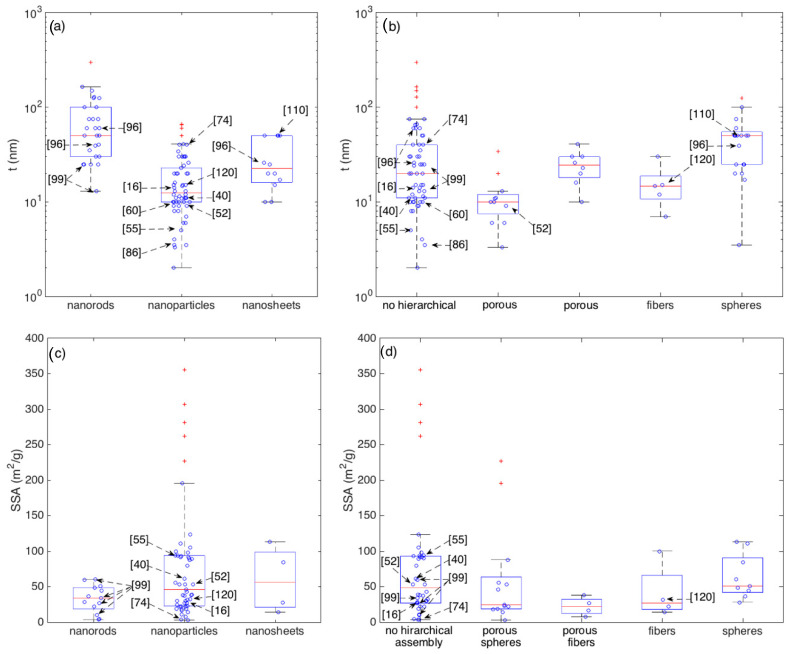
Boxplots showing the statistics of the crystallite thickness (t) grouped according to crystallite morphologies (**a**) and their eventual hierarchical assembly (**b**) and the statistics of the Specific Surface Area (SSA) grouped according to crystallite morphologies (**c**) and their eventual hierarchical assembly (**d**).

Figure 8 resumes the statistical distributions of SSA and of t within the crystallite shape and their eventual hierarchical assembly. 

It is interesting to observe that, though some of the outperforming sensors identified in the previous section features small crystallite thickness, there is no a general and clear correlation between smaller thickness and larger responses (outstanding devices), confirming that the crystallite thickness is an important parameter for gas sensing but it may not account for all observations. For example, the nanorods prepared by Kida and coworkers [99] are among the thinnest nanorods but, between the four materials discussed in [99], the most performing one is the thickest. If considered within the category of networks with no hierarchical assembly, all these four nanorod networks feature crystallite diameters within the interquartile range. Other materials, such as those studied in [16,96,110,120] have crystallite sizes close to the median values of their respective classes, though their responses clearly emerge with respect to the median (Figure 5, Figure 6 and Figure 7). 

Comparing the median values, nanoparticles are characterized by a median diameter (t ≈ 13 nm) that is appreciably lower than the ones of nanorods (t ≈ 50 nm) and nanosheets (t ≈ 23 nm). This may be reasonably due to the longer experience gained with nanoparticle-based materials. Indeed, nanostructures with rod-like and sheet-like morphologies have been developed only in recent years and, considering the additional synthesis constrains necessary to achieve such an anisotropic growth, the optimization of the diameter may be not yet optimized as is for nanoparticles.

Concerning SSA, Figure 8c,d show the presence of several SSA outliers, but none of these coincides with outliers of sensor performance. Interestingly, the most responsive layer synthesized in [99] is characterized by an SSA of about 10 m^2^/g, which is close to the lower whisker for both the nanorods and the disordered (no hierarchical assembly) classes.

Another important set of parameters affecting the sensing properties are the size and distribution of pores. As summarized by Equations (7) and (8), large pores ease the diffusion of gas molecules through the sensitive layer hence allowing an optimal exploitation of the whole volume. Indeed, several authors propose these parameters as the key elements underlying the performances of their materials. This is the case of the outstanding nanorod networks developed by Kida and coworkers, whose response to ethanol correlates well with the pore size increase [99]. The porous spheres prepared by Yoon et al. feature a multimodal pore structure, with the three modal material being more performing than the two and single modal counterparts [52]. 

**Figure 9:**
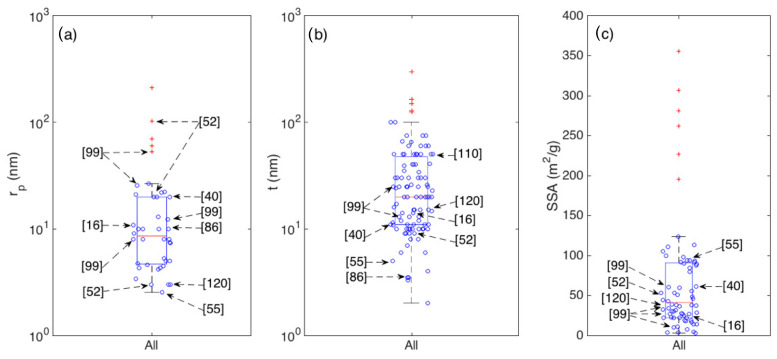
Boxplots showing the statistics of average pore radius r_p_ (**a**); crystallite thickness t (**b**); and Specific Surface Area SSA (**c**), for the whole set of considered articles.

Unfortunately, the pore distribution is not systematically investigated in literature, hence its statistical analysis is harder than it is for crystallite thickness and SSA. Due to the reduced number of papers reporting the pore diameter of analysis, the related boxplot is reported in Figure 9a for the whole set of samples, with no distinction between morphological classes. To compare it with the other main parameters discussed above (t and the SSA), the statistics of these two parameters are also reported in Figure 9b,c respectively.

## 5. Discussion

In this section, the morphological features identified in individual papers as the key properties underlying the observed sensing performances are further discussed with respect to the statistical results outlined in Section 4. The goal is attempting to compare the points of view proposed in individual papers with the point of view that emerges from the statistical analysis. This comparison is organized by subjects in four sub-Sections: 5.1 Crystallite shape effects; 5.2 Surface termination effects; 5.3 Hierarchical organization effects and optimization of gas diffusion; 5.4 SnO_2_ as base material for doped and composite nanostructures. The first three addresses specific morphological features and their effects, the latter focuses on benefits reported for more complex materials, such as those obtained doping of functionalizing SnO_2_, from the morphological features of the base SnO_2_ nanostructures.

### 5.1. Crystallite Shape Effects

According to models summarized in Section 2, nanorods and nanosheets favors the diffusion process with respect to nanoparticles, while spherical crystallites are indicated as the most efficient transducers, followed, in order, by nanowires and finally nanosheets.

At statistical level, the most performing layer is composed by a random network of nanorods [99], on the other hand, most of outliers are composed by nanoparticles [16,40,55,69,86,113,118], confirming the competitive effects intrinsic in the shape of crystallites, but also that all the morphologies offer ample opportunities to achieve outstanding performances.

At the level of individual research papers, some specifically addressed the comparison between different crystallites [51,85,87,96,106]. Interestingly, four out of these five articles, namely [51,85,87,106], reported the response intensity increasing in the following order: nanosheets—nanorods—nanoparticles, which is exactly the opposite trend of the crystallite-shape efficiency predicted in Section 2.3. In this regard, it is worth noting the parallel effects induced by crystallite thickness. Indeed, all four of these papers report t increasing according to the nanosheets—nanorods—nanoparticles order, hence remarking one more time the difficulties in decoupling the effects arising from different structural/morphological features (shape and size in these cases). The only exception with respect to such a response intensity order is provided by Firooz et al. [96]. These authors reported random networks of thin nanosheets (t ≈ 26 nm) being less performing than thicker nanocubes (t ≈ 60 nm and t ≈ 39 nm) organized in random networks and in hierarchical spherical assemblies respectively, with the latter being the most performing. Looking at Figure 5, all three of these layers emerge as competitive with outliers materials [40,120] identified in Figure 6 and Figure 7. 

The comparison of these five articles hence confirms what emerged from the statistical analysis about the difficulties in decoupling the different morphological effects but, at the same time, the large potentialities intrinsic in all the morphologies. 

It is further worth noting that in all these five papers in which different crystallite morphologies are compared, nanoparticles are characterized by larger values of t with respect to their 1D and 2D counterparts, which contrast with the medians extrapolated from the whole dataset (Figure 8a). Reasonably, this difference may arise from the different targets of different authors. In papers addressing the comparison between different morphologies, parameters of the synthesis techniques have been tuned to control the crystallite shape. On papers focused only on nanoparticles, the respective authors better addressed the optimization of t, benefitting from the long-time experience gained with such a traditional morphology, as also observed from a statistical point of view (Figure 8).

### 5.2. Surface Termination Effects

An additional interesting morphological feature is the surface termination. This is a traditional subject in the field of surface science, in which the reactivity of large, single crystals against specific chemicals was studied by means of spectroscopic techniques [131]. Though quite close to gas sensing, a direct application of these findings in this latter field was hindered by the so called pressure-gap and materials-gap. The former accounts for the very different working conditions between gas sensors (room pressure) and spectroscopic techniques (high vacuum or ultra-high vacuum), the latter for the different materials and surfaces used in experiments. Indeed, the large single crystals exposing well defined crystalline surfaces typically employed in surface science feature low performance as gas sensors, on the other hand, the traditional polycrystalline layers composed by rounded nanoparticles used in gas sensing do not feature a clear crystalline termination [131]. In this sense, the advent of single-crystalline nanowires/nanorods/nanocubes and the possibility to develop efficient gas sensors based on these nanostructures offered an important opportunity to reduce such a material gap. Indeed, some recent papers targeted the preparation of faceted, single-crystalline nanostructures with controlled surface termination and their use as gas sensors, showing that high-energy facets such as the (221) plane in SnO_2_ improve the sensing capability [89,92]. Interestingly, the nanorods with this surface termination are the most performing though their diameter, t ≈ 300 nm, which is the largest between the different facet-terminations considered in [92], indicating the effective role played by the surface termination. The response to 100 ppm of ethanol is around 55, compared with responses of about 35, 10 and 10 measured with nanorods exposing (111), (101) and (110) facets and featuring diameters of about 165 nm, 50 nm, and 40 nm respectively. Compared with the statistics of nanorods-based layers (Figure 6 and Appendix A), the four responses reported in [92] are classified in the top 25% (i.e., above Q3), between the top 25% and 50% (i.e., between Q3 and Q2), below Q1 and below Q1 respectively. Unfortunately, the surface termination is still weakly investigated, with several nanorods/nanosheets papers missing this information, and a systematic comparison is not possible. It remains anyway an interesting topic for future studies, especially considering the similar observations about the beneficial effects of high-energy planes reported for other MOX such a ZnO and TiO_2_ [132].

### 5.3. Hierarchical Organization Effects and Optimization of Gas Diffusion

The use of hierarchical assemblies is another method to optimize the diffusion processes. It combines the fine tuning of elementary crystallites at the nanoscale with their hierarchical organization at the μm scale, hence benefitting from the effective transduction enabled by thin elementary nanostructures and the porous structure provided by the hierarchical organization. This method has been exploited with both nanorods, nanosheets and nanoparticles as elementary building blocks. About the latter nanostructures, the hierarchical organization ensures the preservation of an open, porous structure, even in case of close packaging that may occur at the level of adjacent nanoparticles, which is often indicated as a drawback affecting traditional thick films [46]. 

Considering nanoparticle assemblies, it is worth discussing those comparing different hierarchical architectures. For example, a nearly three times improvement (from about 7 to about 20 to 100 ppm of ethanol) is reported for fibers against porous fibers, despite the increase of the nanoparticle thickness from 15 nm to 23 nm, [121]. Wei et al. [83] compared nanoparticles featuring the same dimeter (13 nm) organized in disordered networks and porous spheres, finding an improvement of about 1.5–2 times. The most remarkable example is probably the work published by Yoon et al. [52] who introduced a multimodal porous structure in hierarchical spheres to enhance the capability to detect ethanol. More in detail, the response to 5 ppm increased by about one order of magnitude, from 34 to 118 and 316, by changing the hierarchical structure from dense spheres to porous spheres with bimodal porosity (3 nm and 100 nm) and finally to porous spheres with a trimodal porosity (3, 20 and 100 nm). In this work, crystallites composing the dense spheres were the smallest (t ≈ 6 nm), while nanoparticles of porous spheres were slightly larger (t ≈ 9 nm for both modal distributions). The trimodal pore distribution, spanning over the scales of the micro-, meso- and macro-porosity was proposed as the key feature for the most performing material [52], which emerge as a remarkable outlier from a statistical point of view in Figure 7. 

Methods to optimize the gas-diffusion process have also been developed with disordered networks of nanoparticles. For example, Tan et al. used the target molecule itself to imprint a target-tuned pore size [59]. Comparing 4 samples, the improvement is by a factor of about 4, with the response to 50 ppm of ethanol increasing from 4 to 15, with optimal pore size around 4.5 nm. The fine tuning of mesopores was also studied in [63], observing the response increasing from 3 to 15 with increasing the pore size from 3 to 5.3 nm. An alternative approach was adopted by Tricoli and Pratsinis [60], who achieved remarkable properties, emerging as outliers in Figure 7, by means of a disordered network of thin nanoparticles (t ≈ 10 nm), in which the optimal exposure to the gas molecules through the whole volume of the film is obtained by strongly reducing the film thickness (≈100 nm). Though this configuration do not matches the structure of the traditional thick film, which usually feature a thickness of exceeding the μm, it share with the thick film the granular and porous morphology, with electrical transport occurring through the random network of well-defined elementary units [14]. 

### 5.4. SnO_2_ as Base Material for Doped and Composite Nanostructures

Before to conclude, it is worth highlighting how the development of an efficient SnO_2_ layer is often used as the starting point to further tune the sensing properties of the base SnO_2_ material through addition of dopants or the surface functionalization with suitable nanostructures. The beneficial morphological features of the base SnO_2_ material are often indicated among the key features underlying the remarkable performance observed also with the doped and composite materials.

For example, this is the case of the Al-doped SnO_2_ layers studied by Suematsu et al. [16], in which Al-doping was used to promote a lower cross-sensitivity to humidity, though payed with a decreased response intensity with respect to the pristine SnO_2_ material. Thanks to the outstanding sensitivity of the base material, which emerges as an outlier in Figure 6, the doped material exhibited anyway a remarkable response intensity to ethanol. Similarly, highly performing hollow spheres were further functionalized with Pt and Rh nanoparticles to optimize the sensitivity of the final material to ethanol [113] and formaldehyde [118] respectively. In both cases, the sensing performances were ascribed to both the positive effects of the metallic nanoclusters and the finely controlled structure of the base SnO_2_ hollow spheres. From a statistical point of view, they both emerges as competing well with outliers, as detailed in Section 4.1. Spherical assemblies of nanosheets were used as the base material to develop a SnO_2_ layer functionalized with Au nanoparticles that would benefit from the catalytic Au activity and from the suitable structure achieved by combining sheet-shaped crystallites and their hierarchical spherical assembly [109]. The response of the base material (≈43 to 100 ppm) is quite close to the Q3 value of both the nanosheet and the hierarchical spheres statistics. The reduced grain size (≈10 nm) of the SnO_2_ nanoparticles was also indicated as a key feature, in combination with Ga_2_O_3_ functionalization, for the enhanced sensitivity of the proposed composite material to ethanol [69]. In particular, the response of the base SnO_2_ (≈2000 to 300 ppm) is comparable with the values reported by the outlier-materials developed by Firooz et al. [96]. Hollow fibers were used as well. This is the case, for example, of nanoparticles featuring such a hierarchical organization that were further functionalized with graphene oxide [125] or doped with Yb [54]. In both case, the response intensities (≈69 and ≈71 to 100 ppm of ethanol) of the base hollow fibers lies in the top 25% of both nanoparticles and porous fiber statistics (Figure 6 and Appendix A). 

## 6. Conclusions

This paper reviews the results obtained in the last five years with chemiresistors based on pure SnO_2_ against ethanol vapors in a dry air background. The aim is to identify the central performance of these gas sensors and the presence of any remarkable outlier.

The statistical analysis is carried out grouping the materials according to their morphology, both at the level of elementary building blocks and at the level of their eventual hierarchical assembly. 

What most emerges from the analysis are a few, individual materials outperforming the rest of the dataset, while, in terms of central performance, there is no clear evidence for any morphology working better than others. Overall, the general impression is that disordered assemblies of nanoparticles (the traditional thick film), though in principle less effective than other morphologies, offers anyway the possibility to tune the film features in such a way to compete well with other morphologies. Indeed, even if the most sensitive materials are based on disordered networks of nanorods [99], several outliers are based on the thick film configuration [16,40,55,60,69,74,86,120]. Comparable sensing capabilities have also been reported for a few other materials, namely the nanoparticles organized in porous spheres assemblies with multimodal pore structure reported by Yoon et al. [52], the spherical assemblies of nanorods prepared by Firooz et al. [96], the spherical assemblies of nanosheets developed by Zhou et al. [110], the nanoparticles assembled in hollow spheres reported in [46] and in [118]. 

The retrieved statistical results (outliers and medians) may represent a suitable reference for future work in ethanol sensing, concerning both SnO_2_ and other metal oxides, for which SnO_2_ is often used a benchmarking material. 

In this prospective, a comparison between the point of view obtained from these statistical results and those reported in individual papers has been attempted, discussing in particular the effects arising from the crystallite shape, their surface termination and their hierarchical assembly. Wherever a sufficient statistics was available, both points of view converged in highlighting the complex interplay between the different morphological features and effects, in some cases in competition one another, and the evidence for the broad range of potentialities offered by each morphology, including the traditional thick film, to pursue optimal sensing capabilities. This is further confirmed by a survey on the different SnO_2_ morphologies that have been used as the starting material to develop more complex nanostructures (by doping of SnO_2_ or the use of heterostructures), whose performances were ascribed to both the effective functionalization and the fine characteristics of the base SnO_2_ material.

## Figures and Tables

**Table 1:** Number of different materials (elementary building blocks and their eventual hierarchical organization) whose experimental responses to ethanol have been used for the statistical analysis reported in Section 4. For each morphological type, in addition to the number of materials, the related references are also listed.

Type of Assembly\Building Blocks	Nanoparticles	Nanorods	Nanosheets	Tot.
No hierarchical assembly	Number of materials: 50 References: [16,32,40,50,55,56,57,58,59,60,61,62,63,64,65,66,67,68,69,70,71,72,73,74,75,76,77,78,79,80,81,82,83,84,85,86,87,88,89,90,91]	Number of materials: 18 References: [33,48,87,92,93,94,95,96,97,98,99]	Number of materials: 4 References: [85,87,96,100]	72
Spheres	Number of materials: 4 References: [51,52,85,101]	Number of materials: 11 References: [51,96,102,103,104,105,106,107,108]	Number of materials: 7 References: [49,51,106,109,110,111]	22
Porous spheres	Number of materials: 11 References: [52,83,108,112,113,114,115,116,117,118]	Number of materials: 0	Number of materials: 1 References: [25]	12
Fibers	Number of materials: 7 References: [24,53,91,119,120,121,122]	Number of materials: 0	Number of materials: 0	7
Porous fibers	Number of materials: 8 References: [53,54,121,123,124,125,126]	Number of materials: 0	Number of materials: 0	8
Total	Number of materials: 80	Number of materials: 29	Number of materials: 12	121

## Appendix A

The results of statistical analysis carried out on data shown in the Figure 6a,b boxplots are reported respectively in Table A1 and Table A2.

**Table A1:** Statistical analysis of responses G_gas_/G_air_ to 100 ppm of ethanol grouped by crystallite morphologies, shown in Figure 6a.

	Nanorods	Nanoparticles	Nanosheets
Number of samples	24	51	11
Number of outliers	4	4	2
G_gas_/G_air_, Q1	10.1	11.0	23.0
G_gas_/G_air_, Q2 (median)	25.5	15.8	40.0
G_gas_/G_air_, Q3	51.5	40.8	43.8
G_gas_/G_air_, Whisker low	1.01	1.25	9.2
G_gas_/G_air_, Whisker up	94.0	75.0	44.0
G_gas_/G_air_, arithmetic mean	5479	182	45
G_gas_/G_air_, std	20,465	571	34
G_gas_/G_air_, skewness	4.4	3.6	1.4
*p*-value Kolmogorov-Smirnov test	0.0001	8 × 10^−11^	0.13
*p*-value median test, nanorods	NaN	0.12	0.05
*p*-value median test, nanoparticles	0.12	NaN	0.02
*p*-value median test, nanosheets	0.05	0.02	NaN

**Table A2:** Statistical analysis of responses G_gas_/G_air_ to 100 ppm of ethanol grouped by crystallite assembly shown in Figure 6b.

	No Hierarchical Assembly	Porous Spheres	Fibers	Porous Fibers	Spheres
Number of samples	48	7	4	7	20
Number of outliers	7	0	0	1	3
G_gas_/G_air_, Q1	10.0	7.5	9.8	15.0	14.5
G_gas_/G_air_, Q2 (median)	18.8	14.0	12.8	20.4	26.0
G_gas_/G_air_, Q3	47.0	53.2	61.7	61.0	43.2
G_gas_/G_air_, Whisker low	1.0	1.9	7.6	6.5	2.3
G_gas_/G_air_, Whisker up	75.0	65.0	109.9	71.0	48.0
G_gas_/G_air_, arithmetic mean	2916	26	36	34	36
G_gas_/G_air_, std	14,559	27	50	27	32
G_gas_/G_air_, skewness	6.4	0.8	1.2	0.6	1.6
*p*-value Kolmogorov-Smirnov test	4 × 10^−8^	0.67	0.37	0.64	0.27
*p*-value median test, no hierarchical assembly	NaN	0.72	0.30	0.65	0.29
*p*-value median test, porous spheres	0.72	NaN	0.30	0.59	0.23
*p*-value median test, fibers	0.30	0.30	NaN	0.30	0.27
*p*-value median test, porous fibers	0.65	0.59	0.30	NaN	0.92
*p*-value mediantest, spheres	0.29	0.23	0.27	0.92	NaN
